# Successful treatment of secondary poor graft function post allogeneic hematopoietic stem cell transplantation with eltrombopag

**DOI:** 10.1186/s13045-018-0649-6

**Published:** 2018-08-16

**Authors:** Cen Tang, Feng Chen, Danqing Kong, Qinfen Ma, Haiping Dai, Jia Yin, Zheng Li, Jia Chen, Xiaming Zhu, Xinliang Mao, Depei Wu, Xiaowen Tang

**Affiliations:** 1 Department of Hematology, The First Affiliated Hospital of Soochow University, Jiangsu Institute of Hematology, Suzhou, China; 2 Institute of Blood and Marrow Transplantation, Suzhou, China; 3 Collaborative Innovation Center of Hematology, Soochow University, Suzhou, China; 4 Department of Pharmacology, College of Pharmaceutical Sciences, Soochow University, Suzhou, Jiangsu China; 5 Institute of Clinical Pharmacology, Guangzhou University of Chinese Medicine, 12 Jichang Road, Baiyun District, Guangzhou, 510405 China

**Keywords:** Eltrombopag, Secondary poor graft function, Allogeneic hematopoietic stem cell transplantation

## Abstract

Poor graft function (PGF) is a life-threatening complication after allogeneic hematopoietic stem cell transplantation (allo-HSCT). Current treatment strategies include the use of growth factors, CD34^+^-selected stem cell boost, mesenchymal stem cell transfusion, and second allo-HSCT, but these treatments are not effective in all patients. Eltrombopag, an oral thrombopoietin receptor agonist, which showed promising results in severe aplasia anemia, may be an alternative choice for PGF patients. Therefore, we treated 12 patients who responded poorly to standard treatments for secondary PGF after allo-HSCT with eltrombopag. The median duration was 116 (35–1000) days from transplantation to PGF diagnosis and 59 (30–180) days from PGF diagnosis to eltrombopag treatment. Eltrombopag was started at a dose of 25 mg/d for 3 days and then increased to 50 or 75 mg/d. Median treatment duration was 8 (2–23) weeks. Ten patients (83.3%) responded to the treatment: 8 achieved complete response (CR), and the remaining 2 achieved partial response. In the 10 responding subjects, median platelet count was 18 (5–27) × 10^9^/L vs 74 (30–117) × 10^9^/L prior to and after treatment. Neutrophil count was 0.51 (0.28–0.69) × 10^9^/L vs 1.84 (0.78–4.90) × 10^9^/L. Hemoglobin was 88 (63–123) vs 101 (78–134) g/L. In the 8 patients who achieved CR, the time from eltrombopag initiation to achieving CR was 29 (10–49) days; the response lasted until the last follow-up in all 8 CR subjects (10–18 months). The 12-month overall survival rate was 83.3%. There was no treatment-related mortality and no evidence of cataract, thrombosis, or any other grade 3/4 toxicities.

Poor graft function (PGF) is a life-threatening complication that occurs in 5–27% of the patients following allogeneic hematopoietic stem cell transplantation (allo-HSCT) [1, 2]. Management strategies, including the use of growth factors [3], CD34^+^-selected stem cell boost [4], mesenchymal stem cell (MSC) transfusion [5], and second allo-HSCT [6], are not effective for all patients.

Eltrombopag, a c-mpl receptor agonist, is an effective treatment for immune thrombocytopenic purpura (ITP) and thrombocytopenia after transplantation [7, 8]. In a recent phase I/II study of 92 patients with severe aplastic anemia (SAA) [9], eltrombopag plus standard immunosuppression resulted in 94% hematological response rate. Considering the similarity between PGF and SAA, we speculated that eltrombopag is also effective against PGF. This retrospective analysis included 12 consecutive patients receiving eltrombopag for secondary PGF after allo-HSCT during a period from February 2016 to October 2017. Secondary PGF (sPGF) was defined as: cytopenia in at least two lineages (platelet < 20 × 10^9^/L, neutrophil < 0.5 × 10^9^/L, hemoglobin < 70 g/L), and/or with transfusion requirements beyond day + 28, with full donor chimerism, without relapse or severe graft versus host disease, and lasting at least for 14 consecutive days [5, 10].

Clinical characteristics of the subjects were summarized in Table 1. All 12 patients responded poorly to previous treatments, including growth factors (*n* = 12), MSCs (*n* = 2), and decitabine (*n* = 2). All but one patient were transfusion-dependent. The median duration was 116 (35–1000) days from transplantation to sPGF diagnosis and 59 (30–180) days from sPGF diagnosis to eltrombopag treatment. Eltrombopag was started at a dose of 25 mg/d for 3 days and then increased to 50 or 75 mg/d. Median duration of eltrombopag treatment was 8 (2–23) weeks. Total dosage was 2487.5 (700–10,500) mg.

**Table 1:** Clinical Characteristics of the 12 sPGF patients

No.	Age	Sex	Underlying disease	Cytopenia	Failed previous treatments (duration)	Eltrom duration, weeks	Total dose of eltrom, mg	Time to CR, days	Best response	Last follow-up
1	21	M	ALL	N, PLT	G-CSF, EPO, TPO, IL-11 PLT transfusion-dependent for 12 months, MSC infusion for 4 times	13	6475	43	CR	Alive
2	25	F	ALL	N, HB, PLT	G-CSF, EPO, TPO, PLT transfusion-dependent for 2 months	2	700	10	CR	Alive
3	35	F	ALL	N, HB, PLT	G-CSF, EPO, TPO, RBC and PLT transfusion-dependent for 2 months	2	700	NA	PR	Dead
4	22	M	ALL	N, HB, PLT	G-CSF, PLT transfusion-dependent for 1 month	8	4200	NA	NR	Dead
5	52	M	AML	N, PLT	G-CSF, IL-11, PLT transfusion-dependent for 1 month	4	700	36	CR	Alive
6	27	F	AML	N, HB, PLT	G-CSF, EPO, TPO, RBC and PLT transfusion-dependent for 3 months	7	1725	NA	PR	Dead
7	53	M	AML	N, PLT	G-CSF, EPO, TPO, PLT transfusion-dependent for 1 month	6	2175	25	CR	Alive
8	42	M	MPAL	N, HB, PLT	G-CSF, EPO, TPO, RBC and PLT transfusion-dependent for 3 months; DAC for 1 course	4	1400	NA	NR	Alive
9	42	F	SAA	N, HB, PLT	G-CSF, PLT transfusion-dependent for 1 month; DAC for 1 course	8	4200	30	CR	Alive
10	29	F	SAA	N, PLT	G-CSF, TPO-dependent for 1 month	23	10,500	28	CR	Alive
11	33	M	SAA	N, PLT	G-CSF, EPO, TPO, PLT transfusion-dependent for 2 months, MSC infusion for 3 times	8	4025	49	CR	Alive
12	47	M	MF	N, PLT	G-CSF, PLT transfusion-dependent for 1 month	8	2800	20	CR	Alive

*M* male, *F* female, *N* neutrophil, *HB* hemoglobin, *PLT* platelet, *sPGF* secondary poor graft function, *AML* acute myeloid leukemia, *ALL* acute lymphocytic leukemia, *MPAL* mixed phenotype acute leukemia, *SAA* severe aplasia anemia, *MF* myelofibrosis, *CR* complete response, *PR* partial response, *NR* no response, *G-CSF* granulocyte colony-stimulating factor, *EPO* erythropoietin, *TPO* thrombopoietin, *MSC* mesenchymal stem cell, *DAC* decitabine, *NA* not available

The overall response rate (ORR) was 83.3% (10/12). Eight patients achieved complete response (CR), as defined by platelet ≥ 50 × 10^9^/L, neutrophil ≥ 1.0 × 10^9^/L, and hemoglobin ≥ 90 g/L, without blood cell transfusion or granulocyte colony stimulating factor for ≥ 7 consecutive days [5]; the time from eltrombopag initiation to achieving CR was 29 (10–49) days. Two patients achieved partial response, as defined by hematopoietic engraftment of at least two lineages (platelet ≥20 × 10^9^/L, neutrophil ≥0.5 × 10^9^/L and hemoglobin ≥70 g/L) but not fulfilling CR criteria.

The follow-up was 18.5 (3–37) months post transplantation. Among the 10 responding patients, median platelet count was 18 (5–27) × 10^9^/L vs 74 (30–117) × 10^9^/L prior to and after treatment (*P* = 0.00008; Fig. 1a). Median neutrophil count was 0.51 (0.28–0.69) × 10^9^/L vs 1.84 (0.78–4.90) × 10^9^/L (*P* = 0.0015; Fig. 1b). Median hemoglobin was 88 (63–123) vs 101 (78–134) g/L (*P* = 0.0001; Fig. 1c). The response lasted to the last follow-up (10–18 months) in all 8 subjects who achieved CR.

**Fig. 1:**
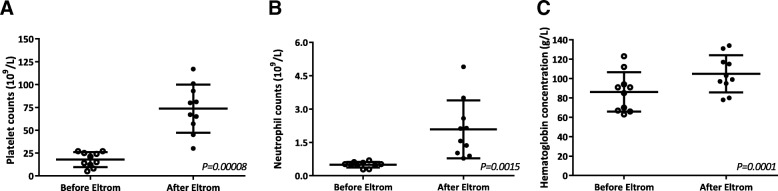
Blood cell counts prior to and after eltrombopag treatment. The analysis included only 10 responding patients. **a** Median platelet count was 18 (5–27) × 10^9^/L vs 74 (30–117) × 10^9^/L before and after the treatment (*P* = 0.00008). **b** Median neutrophil count was 0.51 (0.28–0.69) × 10^9^/L vs 1.84 (0.78–4.90) × 10^9^/L (*P* = 0.0015). **c** Median hemoglobin level was 88 (63–123) vs 101 (78–134) g/L (*P* = 0.0001)

Eltrombopag was well tolerated by all 12 patients. There were no treatment-related mortality and no evidence of cataract, thrombosis, or any other grade 3/4 toxicities. Upon the last follow-up, 9 subjects were PGF-free; 9 had normal blood cell counts. The 12-month overall survival rate after transplantation was 83.3% (95% CI: 62–100%).

With increasing application of alternative donor in transplantation, especially haploidentical HSCT and cord blood transplantation, PGF has become a major obstacle contributing to higher non-relapse mortality. Eltrombopag, as a stimulator of platelet production, promotes the proliferation of megakaryocytes by binding with thrombopoietin receptor (c-mpl) [7], also can promote hematopoiesis along all three lineages. Indeed, clinical trials have establishedefficacy of eltrombopag against ITP, thrombocytopenia after transplantation, as well as SAA [7–9]. Considering the fact that all patients in the current study failed previous treatments for sPGF, the ORR (83.3%) and CR (66.7%) are encouraging. Another important finding is the relatively long duration of the response after eltrombopag withdrawal. The current study represents the first case series of using eltrombopag for secondary PGF after allo-HSCT. Due to the retrospective nature of the study and the small sample size, the results must be considered preliminary and should be verified by randomized controlled trials in the future.

In summary, we showed that eltrombopag could produce a rapid and sustaining response in patients with sPGF after allo-HSCT who failed treatment with conventional treatments. This finding is particularly interesting considering the increasing use of alternative donor HSCT and high rate of non-relapse mortality due to PGF.

## Abbreviations

allo-HSCT: Allogeneic hematopoietic stem cell transplantation
CR: Complete response
ITP: Immune thrombocytopenic purpura
MSC: Mesenchymal stem cell
ORR: Overall response rate
PGF: Poor graft function
SAA: Severe aplastic anemia
sPGF: Secondary PGF
